# Complete genome sequencing and genomic characterization of two *Escherichia coli* strains co-producing MCR-1 and NDM-1 from bloodstream infection

**DOI:** 10.1038/s41598-017-18273-2

**Published:** 2017-12-20

**Authors:** Beiwen Zheng, Xiao Yu, Hao Xu, Lihua Guo, Jing Zhang, Chen Huang, Ping Shen, Xiawei Jiang, Yonghong Xiao, Lanjuan Li

**Affiliations:** 1 Collaborative Innovation Center for Diagnosis and Treatment of Infectious Diseases, State Key Laboratory for Diagnosis and Treatment of Infectious Diseases, The First Affiliated Hospital, College of Medicine, Zhejiang University, Hangzhou, China; 2 Department of Respiratory Diseases, The First Affiliated Hospital, College of Medicine, Zhejiang University, Hangzhou, China; 3 College of Basic Medical Sciences, Zhejiang Chinese Medical University, Hangzhou, China

## Abstract

We previously described the discovery of two *Escherichia coli* isolates (EC1002 and EC2474) co-harbouring *mcr-1* and *bla*_NDM-1_ genes, which were recovered from bloodstream infection in China. More importantly, these antibiotic resistance genes were located on different plasmids and signaling the potential spread of pandrug-resistant bacteria. Here, the complete genome sequences of both isolates were determined using Pacbio RS II and Illumina HiSeq2000 systems. The genome of EC1002 consists of a 5,177,501 base pair chromosome and four circular plasmids, while the genome of EC2474 consists of a 5,013,813 base pair chromosome and three plasmids. The plasmid replicon type of pEC1002_NDM and pEC2474_NDM were identified as IncA/C2 and IncF, respectively. The genetic environment of *bla*_NDM-1_ in this study was similar to *bla*_NDM_-carrying plasmids detected in China, although the overall nucleotide identity and query coverage were variable. The plasmid replicon type of pEC1002_MCR and pEC2474_MCR were identified as IncI2 and IncHI2, respectively. Two different genetic strategies for *mcr-1* gene spread were observed in this study and *bla*_NDM-1_ genes were also found transferred by two different mobile genetic elements in two plasmids. The findings of this study further support that the diversified transfer mechanisms of *bla*_NDM-1_ and *mcr-1* present in *Enterobacteriaceae*.

## Introduction

The increasing prevalence and dissemination of carbapenemase-producing *Enterobacteriaceae* (CPE) is a worldwide public health issue^1,2^. Recently, CPE were listed as the most critical group of pathogens by the World Health Organization^3^. The New Delhi metallo-β-lactamase (NDM) is one of the most common carbapenemases worldwide. Colistin is an antibiotic often referred to as a “last resort” for the treatment of CPE infections^4^.

Recently, concerns were raised regarding the increasing prevalence of the first plasmid-mediated colistin resistance gene, *mcr-1*, which was identified in animal and human food sources in China^5^. Subsequently, this transmissible gene has been detected in many countries^6–12^. The emergence of *mcr-1* further narrows clinical therapeutic options, which is a potential concern to public health. Furthermore, *mcr-1*-harboring strains have been isolated from bloodstream infections (BSI) in China^13,14^. Altogether, this brings serious health hazards, particularly if *mcr-1* carrying isolates continue to spread in clinical settings^14–16^.

So far, several separate groups reported the co-occurrence of MCR-1 and NDM-1 on plasmids in *Enterobacteriaceae* ^17,18^. Furthermore, we have reported the isolation of two *Escherichia coli* strains from BSI, which harbor the *bla*_NDM-1_, *mcr-1*, and *bla*_CTX-M_ genes^13^. More importantly, these antibiotic resistance genes were located on different plasmids and signaling the potential spread of pan-drug-resistant bacteria. However, genomic hallmarks of the bacterial host reservoir for carbapenemase-producing and *mcr-1*-encoding plasmids remain unclear. In this study, we investigated the genetic features of these two isolates and elaborated on various potential mechanisms by which *mcr-1* and *bla*_NDM-1_ may be transmitted. In addition, comparative analyses of the genetic contexts of *mcr-1* and *bla*_NDM-1_ with closely related plasmids were also performed.

## Materials and Methods

### Bacterial isolation and genome sequencing

*E. coli* EC1002 and EC2472 carrying both *bla*_NDM-1_ and *mcr-1* were isolated from BSI patients in the Affiliated Hospital of Jining Medical University and Anhui Provincial Hospital, respectively^13^. Genomic DNA was extracted from overnight cultures using a Gentra Puregene Yeast/Bact. Kit (Qiaqen, Hilden, Germany) according to the manufacturer’s instructions. The harvested DNA was visualized on 1% (w/v) agarose gels, and DNA concentration as well as purity was determined by a NanoDrop 2000 UV-Vis Spectrophotometer (Thermo Scientific, Waltham, MA, USA) and Qubit®2.0 Fluorometer (Thermo Scientific, Waltham, MA, USA). DNA was stored at −20 °C until further processing. The genome of the two isolates was sequenced using the Pacbio RS II (Pacific Biosciences, Menlo Park, CA, USA) and Illumina HiSeq. 2500-PE150 platform (Illumina, San Diego, CA, USA). A 10-kb DNA library was constructed by the PacBio SMRTbell 10 kb Library preparation kit according to the manufacturer’s instructions (Pacific Biosciences, Menlo Park, CA, USA). Pair-end index libraries construction followed the NEBNext Ultra DNA Library Prep Kit (Illumina, SanDiego, CA, USA). Library construction and sequencing was performed at Beijing Novogene Bioinformatics Technology Co. Ltd.

### Genome Assembly

Low quality reads were filtered out and the filtered reads were assembled to generate one contig without gaps by SMRT 2.3.0 using Hierchical Genome Assembly Process (HGAP) V.3.0. Overlaping regions were assessed with Gepard followed by circularization using minimus2 pipeline in the AMOS software package^19^. Subsequently, Illumina HiSeq contigs were mapped over the PacBio-generated contigs to correct the assembled contigs.

### Genome annotation, and *in silico* analyses

Protein-coding genes were initially identified and annotated using RAST^20^ and further annotated by BLASTP against UniPort and NR databases, while insertion elements (IS) were identified using IS Finder^21^. Queries were generated using the ResFinder 2.1 database to identify acquired antibiotic resistance genes^22^. Plasmid Finder 1.3 and pMLST were used to identify plasmid incompatibility types^23^. The circular image and circular comparisons between multiple genomes and plasmids was done by BLAST Ring Image Generator (BRIG)^24,25^. Linear comparison figures of multiple plasmids were generated by a Python application Easyfig.^26^.

### Nucleotide sequence accession numbers

The complete sequences of *E. coli* EC1002, EC2474, and other plasmids have been deposited in GenBank under the accession numbers CP021202-CP021210 (Table 1).

**Table 1:** Genomic features of EC1002 and EC2474.

**Chromosome/plasmid**	**Plasmid replicon type**	**Size (bp)**	**GC content (%)**	**Accession numbers**	**CDS**	**Resistance genes** ^**a**^
EC1002	—	5,177,501	50.1	CP021202	4,808	*bla*_CTX-M-15,_ *oqxB, tetB*,
pEC1002-1	IncFII	183,509	50	CP021203	234	*bla*_CTX-M-15,_ *sul, mph*, *aac(3)-Ib, erm, aadA4, dfrA, arr*
pEC1002-MCR	IncI2	63,392	43	CP021205	97	*mcr-1*
pEC1002-NDM	IncA/C2	111,688	52.3	CP021206	154	*bla*_NDM-1_, *bla*_CTX-M-14_, *bla*_TEM,_ *sul1*, *mph*, *aac(6′)-Ib, rmtc, arr*
pEC1002-4	IncFIB	92,439	50	CP021204	136	*bla*_TEM_
EC2474	—	5,013,813	50.6	CP021207	4,938	*bla*_CTX-M-55_
pEC2474-MCR	IncHI2	223,982	45.8	CP021209	284	*mcr-1*, *bla*_CTX-M-14,_ *floR, aph4, sul2, aac(3)-IVa, fosA14*
pEC2474-NDM	IncFII	75,553	50.8	CP021210	110	*bla*_NDM-1_, *aph*
pEC2474-3	IncI1	86,717	49.5	CP021208	124	*bla*_CTX-M-55_

^a^aac(3)-*IB*: aminoglycoside acetyltransferase-*IB*; aad: aminoglycoside adenylyltransferase; *adrA*: dihydrofolate reductase; *aph*: aminoglycoside-3′-O-phosphotransferase; *arr*: rifampin ADP-ribosylating transferase; *erm*: erythromycin resistance methylase; *oqxB*: olaquindox resistance; *tetB*: tetracycline resistance; *fosA*: glutathione transferase; *mph*: macrolide phosphotransferases; *rmtC:* ribosomal RNA methyltransferase; *sul*: sulphonamide resistance.

## Results and Discussion

### Basic genomic features

**Figure 1:**
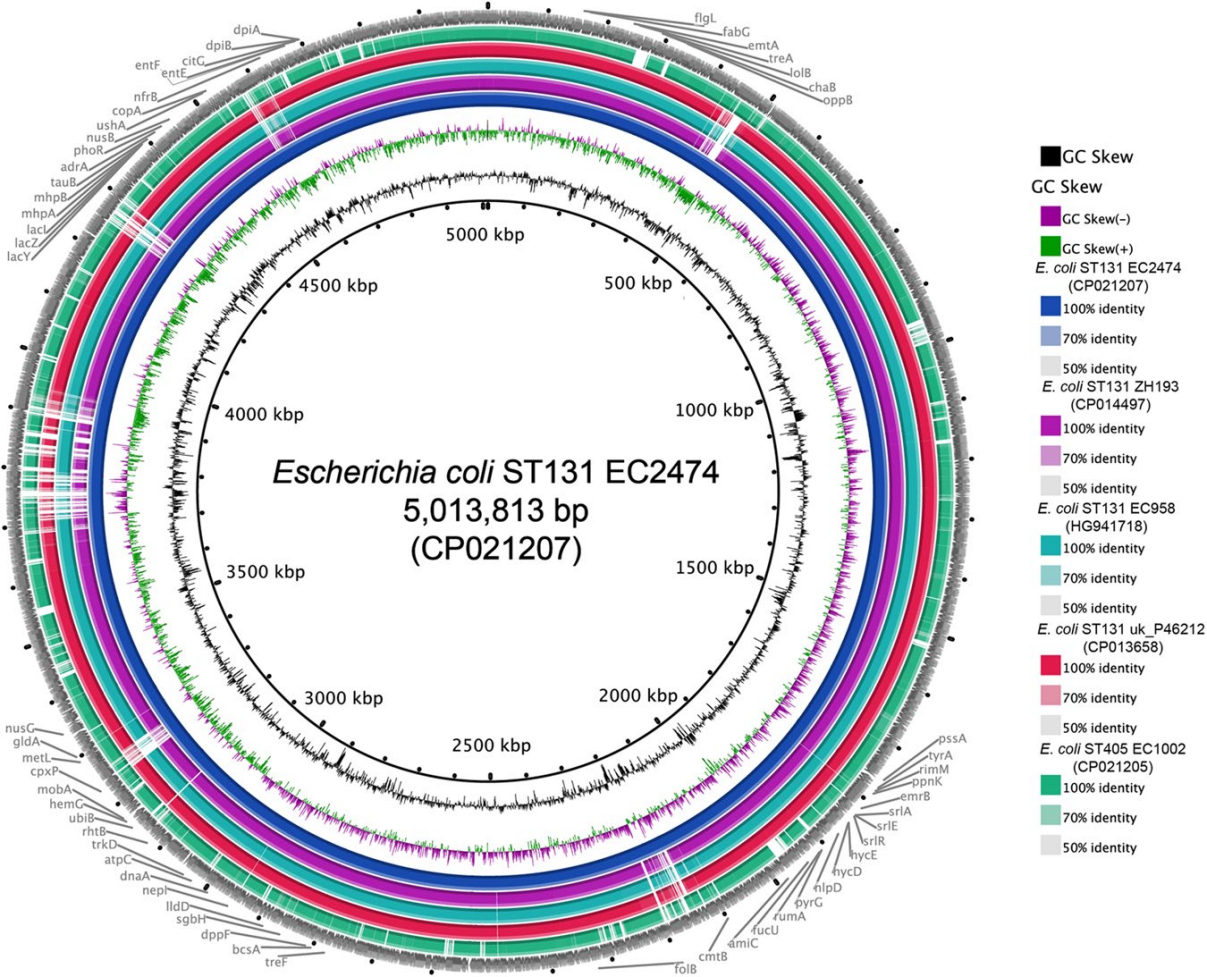
Circular map of chromosomes from EC1002, EC2474, and three related isolates. GC content and GC Skew are represented on the distance scale (in kbp) on the inner map. The arrows around the map indicate deduced ORFs and their orientation. EC2472 (CP021207), ZH193 (CP014497), ZH063 (CP014522), and uk_P46212 (CP013658) were isolated from *E. coli* ST131, while EC1002 (CP021202) was isolated from *E. coli* ST405.

The genomic features and a comparison of EC1002 and EC2474 against other *E. coli* isolates are summarized in Fig. 1 and Table 1. All plasmids were assembled into a circular ring and the chromosome was assembled into one contig. It was determined that the genome of EC1002 consists of a 5,177,501 base pair chromosome with an average 50.1% GC content and four circular plasmids, while EC2474 consists of a 5,013,813 base pair chromosome with an average 50.6% GC content and three plasmids. Screening for acquired resistance determinants revealed the presence of different kinds of resistance genes (Table 1). The isolates EC1002 and EC2474 belonged to ST405 and ST131, respectively. *E. coli* sequence type 131 (ST131) is a worldwide pandemic clone, causing predominantly community-onset antimicrobial-resistant infection and subsequent study has confirmed the worldwide prevalence of ST131 harbouring a broad range of virulence and resistance genes on a transferable plasmid, while ST405, which is an high risk clone found in human, animals and environment usually associated with CTX-M-types^27,28^. At present, several different *E. coli* ST isolates such as ST167, ST206, ST648, and ST156 have also been reported to carry both *bla*_NDM_ variants and *mcr* genes^29–31^. Interestingly, a recent study observed significant geographical clustering with regional spread of *mcr-1*-bearing IncHI2 plasmids in Europe and IncI2 in Asia^32^. The unrelated clonally relationship found in this study suggesting coexist these two plasmids could also be horizontal transferred to other STs. Furthermore, the detection of florfenicol resistance gene*, floR*, in the genome of isolate EC2474 together with the fact that florfenicol is widely used in veterinary medicine further supports the potential transfer of *mcr-1* gene from animals to clinical settings^32^.

### Genetic characteristics of plasmids bearing *mcr-1*

The GC content of *mcr-1* bearing plasmids in this study was similar to that of previously reported *mcr*-bearing plasmids^33^. However, they were also found to be significantly different to other plasmids exist in the same strain (Table 1). pEC1002-MCR is a 63,392 bp circular plasmid encoding the IncI2 replication protein. In contrast, pEC2474-MCR is a 223,982 bp IncHI2 plasmid. The IncI2-type plasmid is considered to be a major genetic event driving the rapid mobilization and acquisition of *mcr* genes^34^. The IncHI2-type plasmid is characterized by its long as well as conjugation flexible pilus. Furthermore, the thermosensitivity of the conjugative apparatus means that the optimal temperature for conjugation is 22–30 °C rather than 37 °C^35^. Therefore, this strain is more likely to acquire *mcr*-bearing plasmids *in vitro*, similar to other reports of *mcr*-producing strains, which have been mainly isolated from the agriculture industry in China, indicating environmental origins of *mcr-1* genes in human pathogens^36,37^. pEC1002-MCR only contained the *mcr-1* gene, which is in contrast to other reports where *mcr-1* easily co-exists with other resistance genes^38,39^. However, pEC2472-MCR carried several resistance genes, such as *bla*_CTX-M-14_, *fosA*, and *floR*.

**Figure 2:**
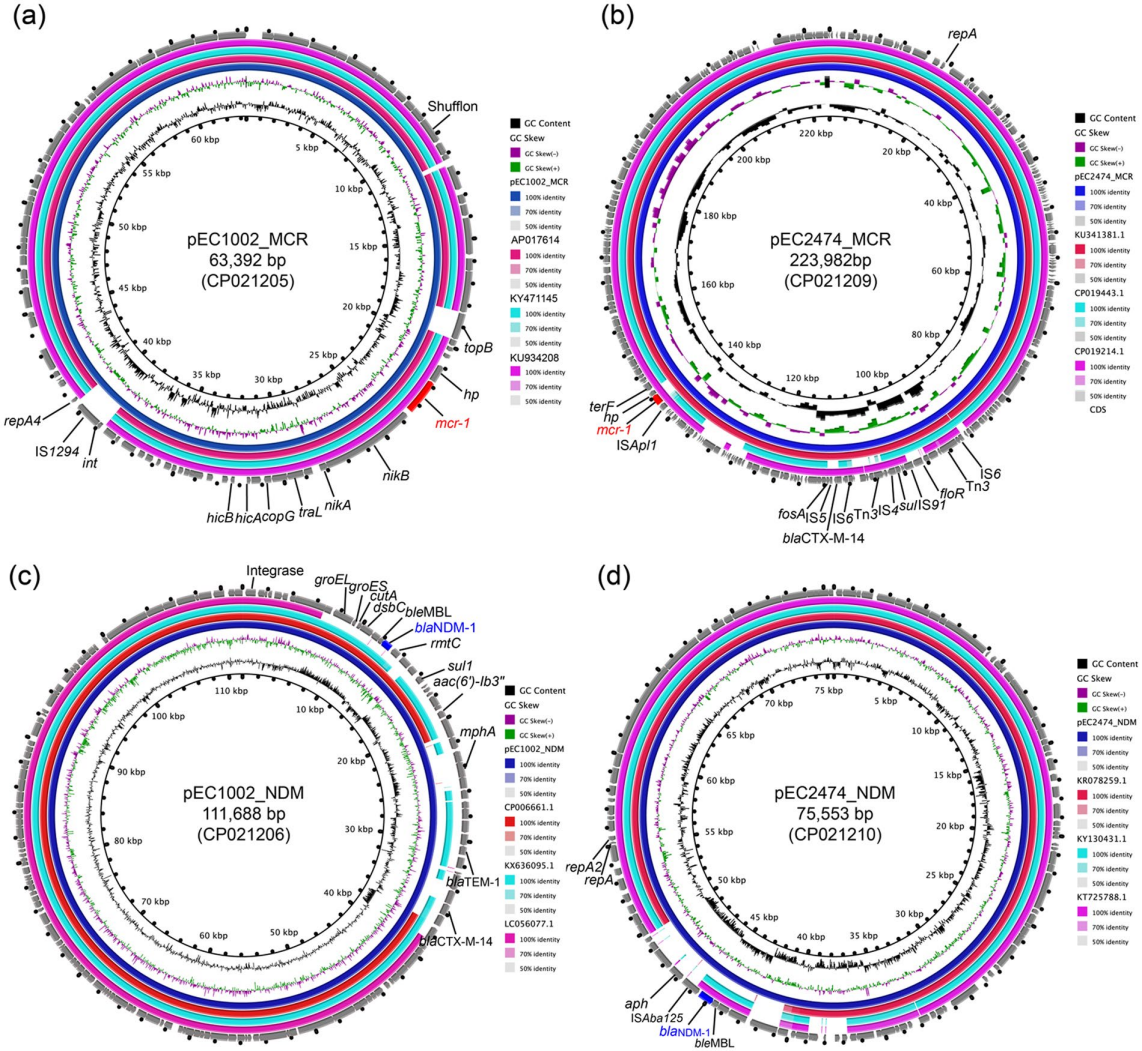
Circular representation of the studied plasmids. GC content and GC Skew are represented on the distance scale (in kbp) on the inner map. Each plasmid was compared to its most closely-related plasmid (Genebank accession numbers shown on the right side). The arrows around the map indicate deduced ORFs and their orientation. Certain important genes are also indicated on the ring. The schematics were generated through the ‘BLAST Ring Image Generator’ (BRIG) program.

**Figure 3:**
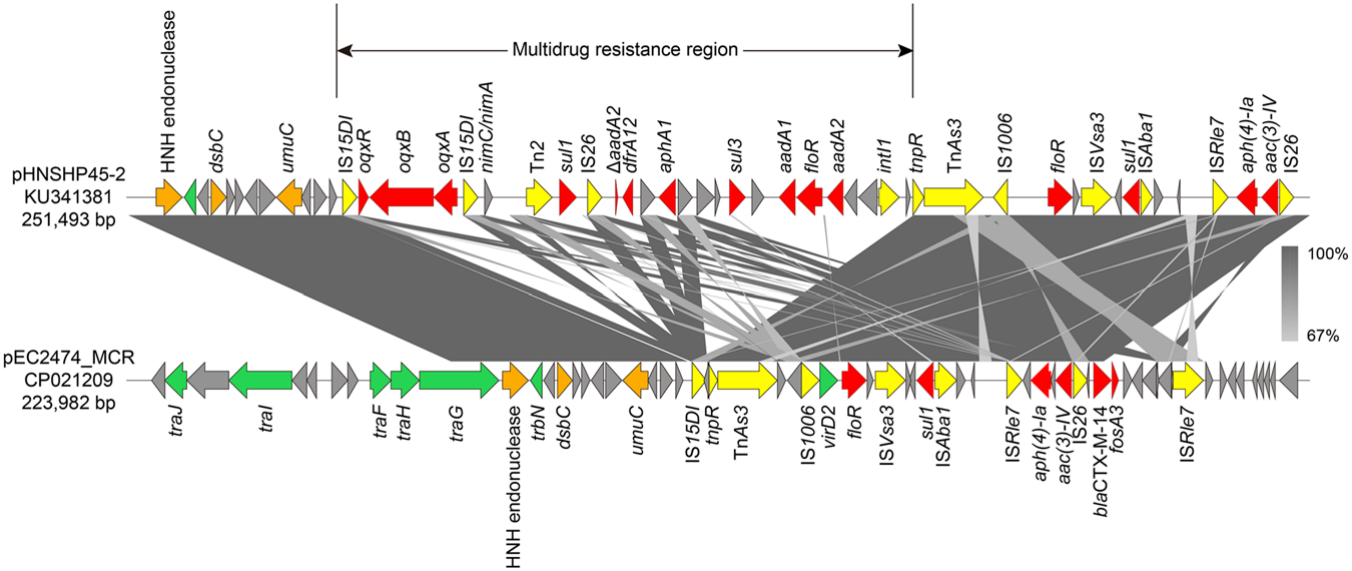
Linear plasmid characterization of pEC2474_MCR with closely related plasmid pHNSHP45-2 (KU341381). The grey regions between plasmids indicate nucleotide identity (65–100%) by BLASTN. Gray shades indicate shared regions with high degree of homology. Arrows indicate predicted open reading frames (ORFs) and colored according to their putative functions. Blue arrows indicate replication associated genes. Yellow arrows indicate conjugal transfer-involved genes. Genes associated with plasmid stability are colored by brown. Antimicrobial resistance genes and mobile elements genes were indicated by red and green arrows, respectively. Grey arrows indicate genes for hypothetical proteins as well as proteins with unknown function.

A BLAST search against the nr/nt database indicated that pEC1002_MCR showed an overall nucleotide identity (99–100%) and query coverage (93–97%) similar to several plasmids, such as pMRY16-002_4 (GenBank no. AP017614)^40^, pHeN867 (KU934208), and pEC019 (KY471145) that have been reported in different countries. In addition, the size and backbone structure of these plasmids are quite similar (Fig. 2a). Further analyses revealed three encoding sequence insertions in pEC1002_MCR. The 18,358–19,666 insertion region and 38,741–40,423 region carrying genes encoding for DNA topoisomerase III (*topB*), integrase (*int*), and IS1294, respectively (Fig. 2a). The region of 10,223–11,517 encodes for shufflon protein A and two shufflon protein C. These proteins are highly mobile DNA segments that function as a biological switch and generally invert independently or in groups resulting in a complex DNA rearrangement. Furthermore, the shufflon rearrangement is closely related to plasmid transmission in *Enterobacteriaceae* ^40^. Of note, the sequence of *nikA-nikB*-*mcr-1*-*hp* region was identified in pEC1002_MCR, which is in contrast to the 2.6 kb *mcr-1*-*pap2* element usually found in *mcr-1-*carrying plasmids^41^. The BLASTN comparison of pEC2474_MCR plasmid found 100% nucleotide identity and 100% coverage with pHNSHP45-2 (KU341381), which is the first reported plasmid carrying *mcr-1*. The main difference between pEC2474_MCR and pHNSHP45-2 is the multidrug resistance region (Fig. 3), suggesting that pHNSHP45-2 may be formed by acquiring an IS region containing several resistance genes.

### Genetic context of plasmids bearing *bla*_NDM-1_

The plasmid replicon type of pEC1002_NDM and pEC2474_NDM were identified as IncA/C2 and IncF, respectively. It has been reported that the IncX3-type plasmid is the main type of NDM-producing plasmid spread in China^42^ and we first reported the NDM-producing IncA/C2 plasmid in mainland China. While pEC1002_NDM carries a variety of drug resistance genes, pEC2472_NDM only carries the *bla*_NDM-1_ and *aph* resistance genes (Table 1).

**Figure 4:**
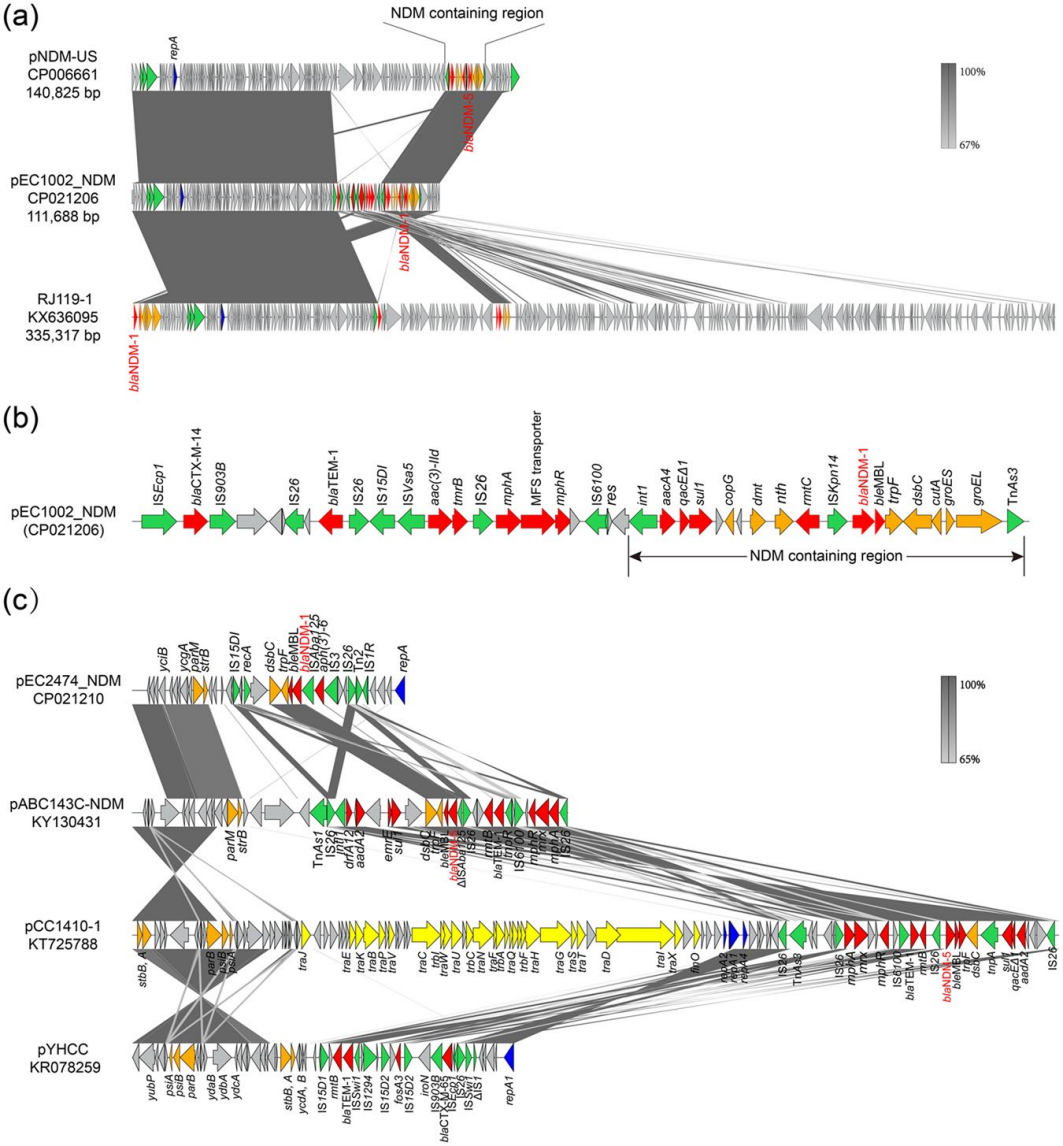
Linear plasmid characterization of NDM-1-bearing plasmids with closely related plasmids. The grey regions between plasmids indicate nucleotide identity (65–100%) by BLASTN. Arrows indicate predicted ORFs. (**a**) Major structural features of pEC1002-NDM compared to plasmids pUS (CP06661) and RJ119-1 (KX636095). (**b**) Schematic representation of the genetic organization surrounding *bla*_NDM-1_ in pEC1002_NDM. ORFs are labeled above the arrows. (**c**) Major structural features of the *bla*_NDM-1_ region in pEC2474-NDM compared to plasmids pABC143C-NDM (KY130431), pCC1410-1 (KT725788), and pYHCC1 (KR078259). ORFs are labeled above the arrows and colored as described in Fig. 1.

BLASTN comparison of the two NDM-1-producing plasmids revealed that the overall structure of both plasmids showed big differences compared to known NDM-1-producing plasmids*. In silico* analyses demonstrated that pEC1002_NDM shared 99% nucleotide identity as well as 71%, 87%, and 93% coverage with pNDM-US from *K. pneumoniae*, pV001-a from *E. coli*, and pRJ119-NDM from *K. pneumoniae*, respectively. Although the overall structure of these plasmids was different, the genetic context of *bla*_NDM-1_ was relatively similar in all plasmids (Fig. 4a), where *bla*_NDM-1_ is located in a mobile region with a structure of *rmtc*-IS*Kpn14*-*bla*_NDM-1_-*ble*_MBL_-*trpF*-*tat*-*dsbC*-*groES*-*groEL*, which is identical to a previous report^43,44^. Notably, the difference between pNDM-US and pEC1002_NDM was the ~17 kb insertion sequence which contains 7 resistance genes, four transposase encoding genes, two resolvase encoding genes, and one integrase encoding gene upstream of *bla*_NDM-1_ (Fig. 4a,b). Interestingly, these resistance genes may originate from different parts of pRJ119-NDM plasmids. As for the pEC2474_NDM plasmid, 99% nucleotide identity was found as well as 89%, 89%, and 87% coverage with plasmid pABC143C-NDM, pCC1410-1, and pYHCC, respectively (Fig. 2d). The backbone structure of these plasmids was similar, except for the region containing *bla*_NDM-1_ (Fig. 4c). The upstream region of *bla*_NDM-1_ encoded a recombinase (*recA*), while a common gene environment around *bla*_NDM-1_ (IS*Aba125*-*bla*_NDM-1_-*ble*_MBL_-*trpF*-*dsbC*) was identified^36,45^. In addition, the region after this structure also contained four mobile genes (Fig. 4c).

## Conclusion

In this study, we report the complete genome sequences of two *E. coli* strains with coexisting genes, *mcr-1* and *bla*_NDM-1_. Two different genetic strategies for *mcr-1* transmission were observed in these two strains. Firstly, the transfer of *mcr-1* associated with a *nikA-nikB*-*mcr-1*-*hp* structure was observed in pEC1002_MCR, while the IS*Apl1*-*mcr-1* mobile element played an important role in pEC2474_MCR. Additionally, a common gene environment around *bla*_NDM-1_ (*rmtc*-IS*Kpn14*-*bla*_NDM-1_-*ble*_MBL_-*trpF*-*tat*-*dsbC*) was detected in pEC1002_NDM, while an IS*Aba125*-*bla*_NDM-1_-*ble*_MBL_-*trpF*-*dsbC* structure was identified in pEC2472_NDM. Taken together, this study further supports that the diversified transfer mechanisms of *bla*_NDM-1_ and *mcr-1* present in *Enterobacteriaceae*.
